# Hypohidrotic Ectodermal Dysplasia: A Case Report

**DOI:** 10.7759/cureus.46530

**Published:** 2023-10-05

**Authors:** Humaira Shamim, Sumera Hanif

**Affiliations:** 1 Dermatology, California Institute of Behavioral Neurosciences & Psychology, Fairfield, USA; 2 Dermatology, College of Physicians and Surgeons, Lahore, PAK; 3 Internal Medicine, Allama Iqbal Medical College, Jinnah Hospital, Lahore, PAK

**Keywords:** finger clubbing, anhidrosis, hidrotic ectodermal dysplasia, ectodermal dysplasia, hypohidrotic ectodermal dysplasia

## Abstract

Ectodermal dysplasia (ED) is a rare genetic disorder that affects the developmental disturbance of ectoderm-derived tissues, organs, and accessory appendages, i.e. skin, hair, tooth, nail, and sweat glands. ED has two types hypohidrotic or anhidrotic ectodermal dysplasia and hidrotic ectodermal dysplasia. We report this case of classical hypohidrotic ectodermal dysplasia (HED) with clubbing. The association of clubbing with HED is still rare. This case report aims to discuss the etiology, clinical manifestations, and management of ectodermal dysplasia. A multidisciplinary approach is required including dentists, nutritionists, dermatologists, and physicians to manage ectodermal dysplasia.

## Introduction

Ectodermal dysplasia is a congenital, non-progressive, and heterogeneous disorder with a wide range of defects in the ectodermal structure [1]. The mode of inheritance of ectodermal dysplasia can be autosomal recessive, autosomal dominant, and X-linked. Some ectodermal dysplasia patients have mild degrees while some have a severe degree with the involvement of hair, skin, nails, and teeth. The other features of ectodermal dysplasia are frontal bossing, sunken cheeks, depressed nasal bridge, low-set ears, and thick lips [1]. Topical moisturizers and emollients can be effective in reducing dry and itchy skin. Orthodontic consultation indicates to ensure good nutritional intake and cosmetic reasons [1-3]. Ectodermal dysplasia is relatively rare with an estimated incidence of seven per 100,000 people [3]. X-linked recessive hypohidrotic ectodermal dysplasia and hidrotic ectodermal dysplasia are the most common types of ectodermal dysplasia [3].

## Case presentation

A 22-year-old male patient presented to the dermatology clinic with complaints of dry skin, no sweating, difficulty tolerating a hot environment, few missing teeth since childhood, and peg-shaped incisors. The patient’s symptoms were noticeable during early childhood when the parents noticed hyperpyrexia, especially in the hot and humid season. On examination of the skin, there was no evidence of sweating and thickened skin on the palms and soles. Scalp and body hair was thin and sparse. Eyebrows showed madarosis. There was no significant history of exertional shortness of breath or other complaints. On general physical examination, pulse was 88 beats per minute, blood pressure was 120/80 mmHg, and oxygen saturation was 98%. Systemic examination was unremarkable. His intelligence was normal and he was well-educated. His family was also educated and aware of this condition and its precautions regarding avoidance of vigorous exercise and exposure to hot environments. He was advised to use emollients for dry skin. He also had dryness in his mouth. He was advised to maintain good oral hygiene, avoid sun exposure, and consult an orthodontic specialist for expert dental management.

**Figure 1:**
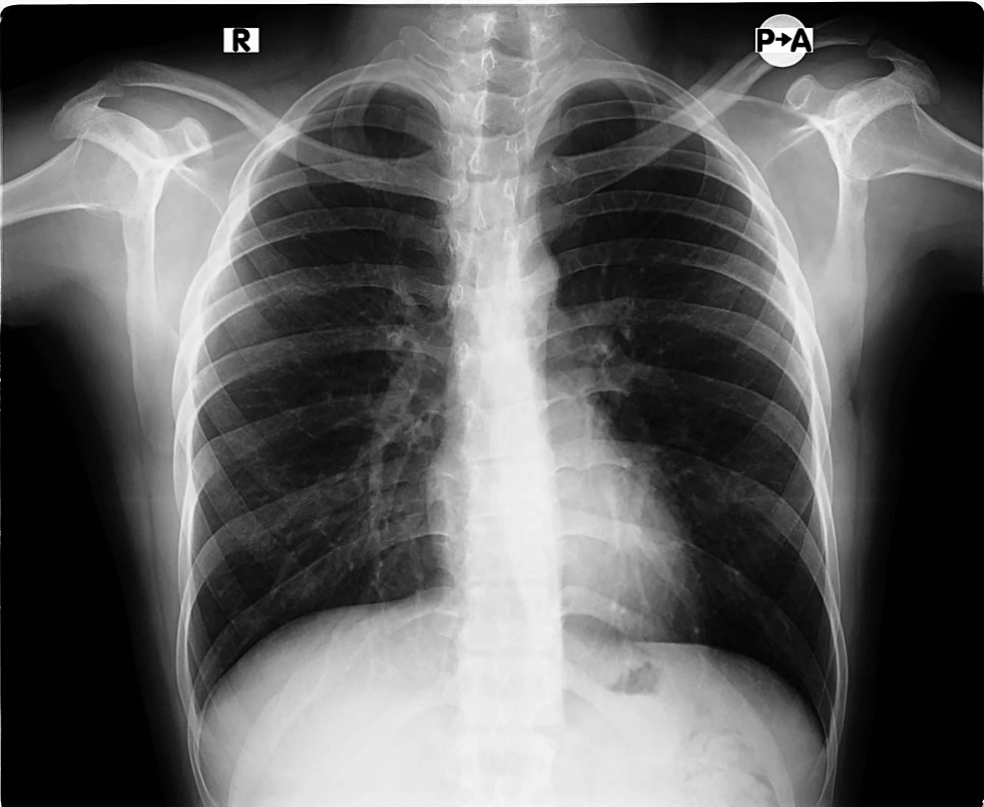
Chest X-ray showing a normal cardiothoracic ratio.

His parents and sister did not have ectodermal dysplasia or any medical issues. However, his brother has decreased sweating and dental issues. There was no history of consanguineous marriage among parents. Baseline laboratory examination and chest X-ray findings were unremarkable with no evidence of congenital heart disease or interstitial lung disease with a normal cardiothoracic ratio. A provisional diagnosis of hypohidrotic ectodermal dysplasia was considered. Differential diagnoses of ectodermal dysplasia, anhidrotic ectodermal dysplasia, and ectodermal dysplasia with clubbing were considered. The chest X-ray of the patient is shown with a normal cardiothoracic ratio in Figure 1.

**Figure 2:**
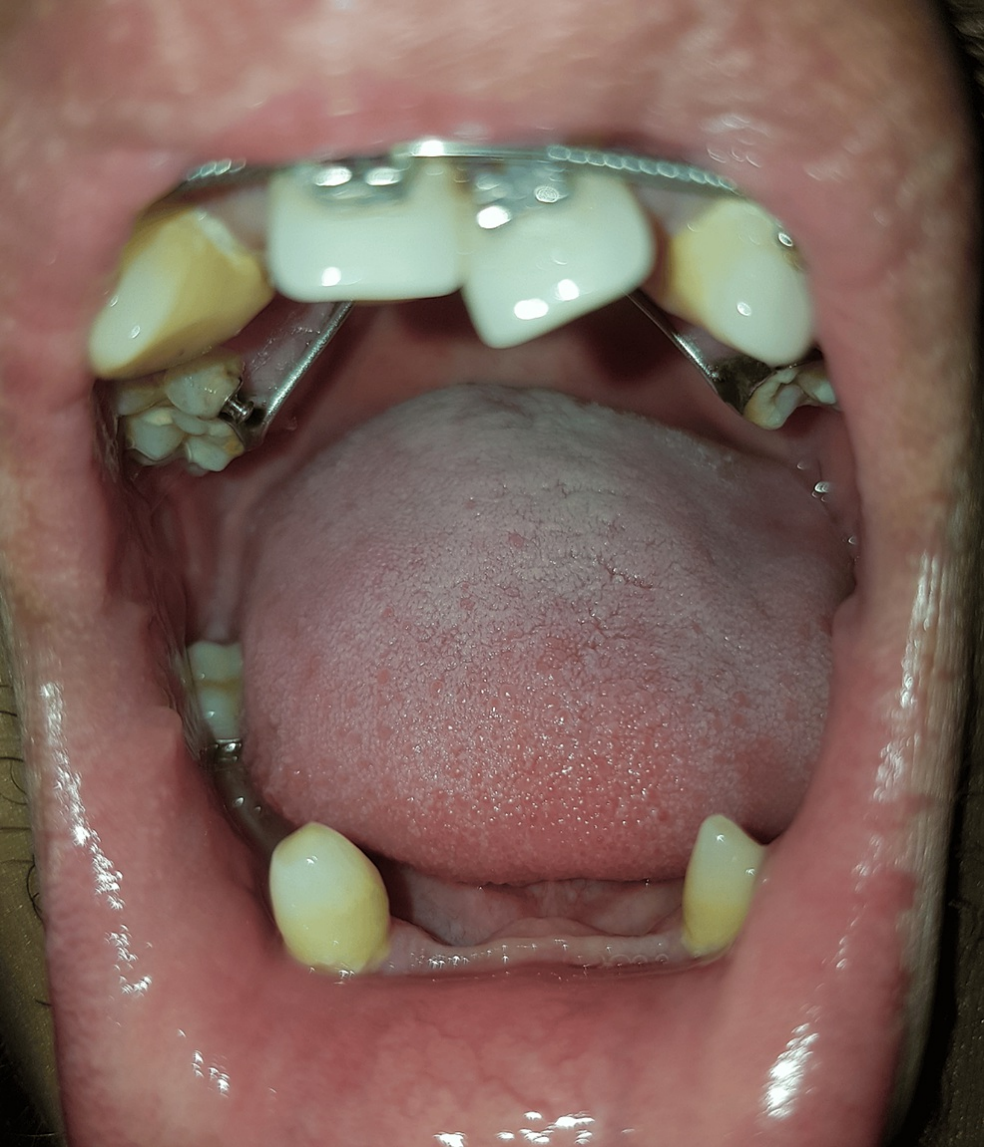
Oral cavity showing missing teeth and peg-shaped teeth.

**Figure 3:**
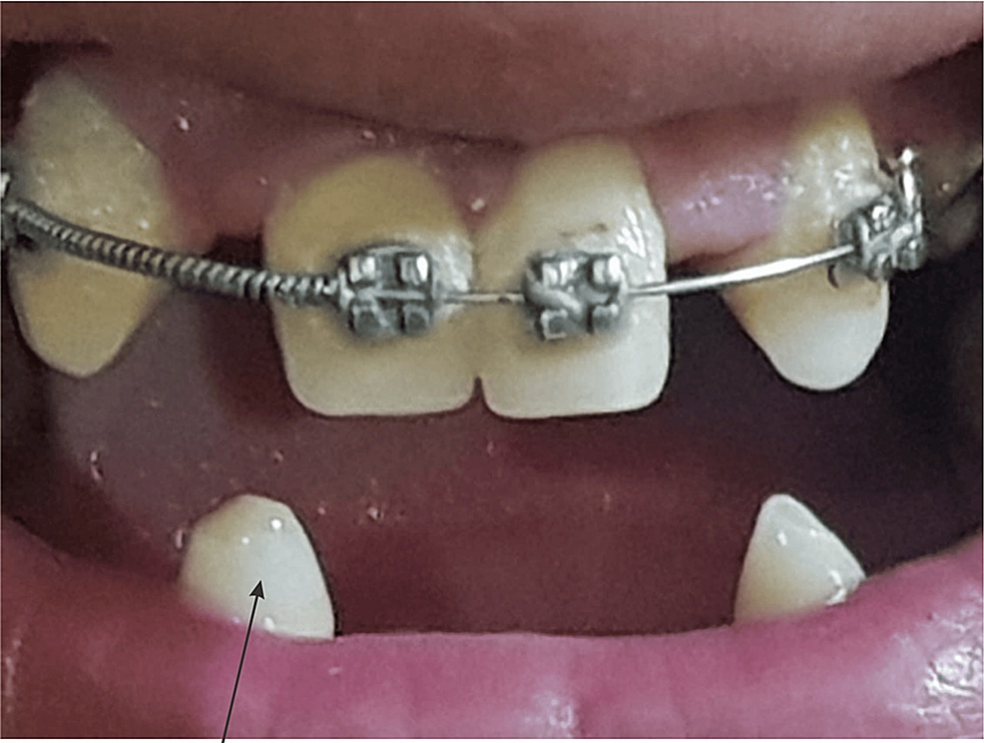
Peg-shaped teeth (demonstrated by arrow) and missing permanent teeth.

**Figure 4:**
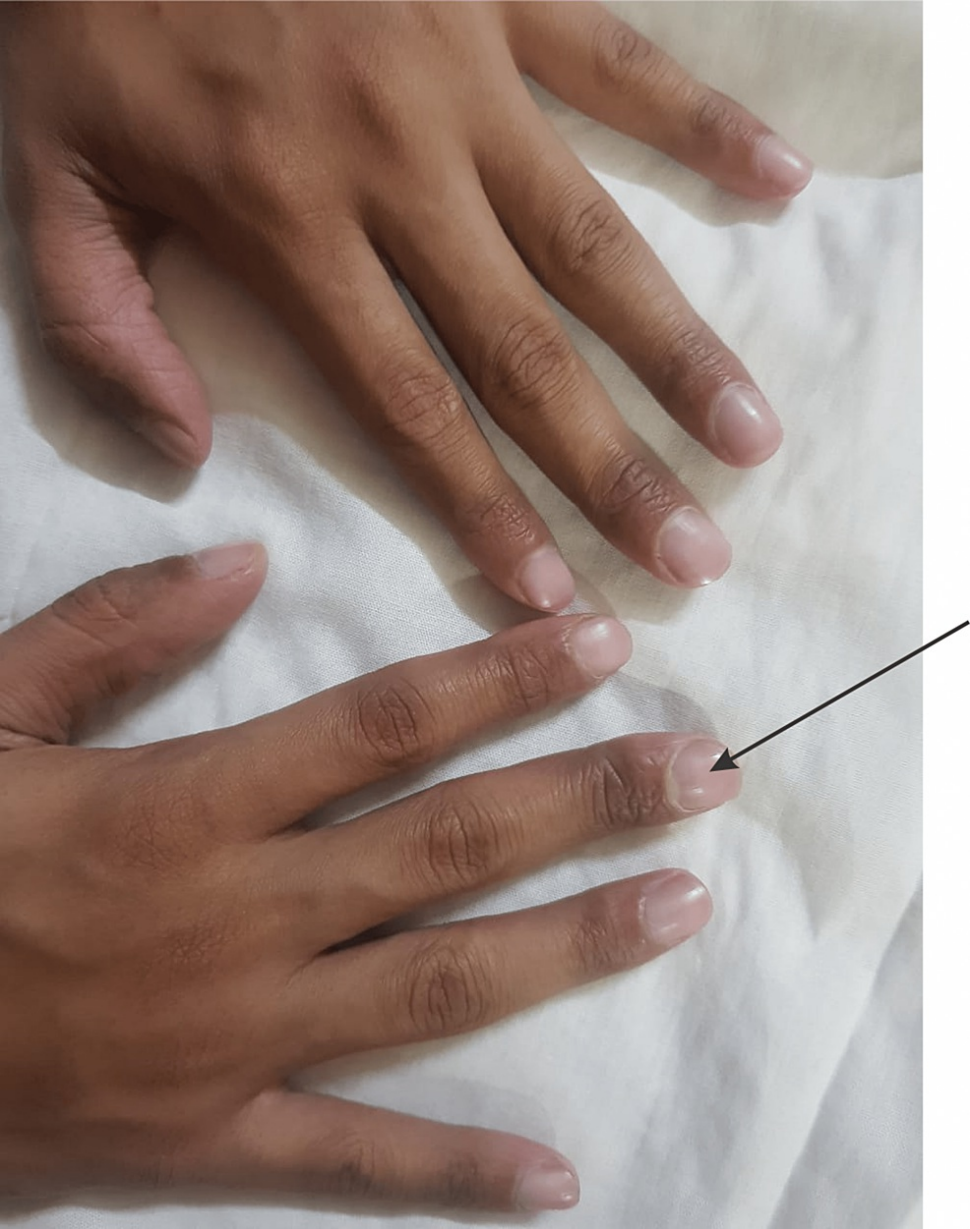
Fingernails of both hands demonstrate clubbing.

The oral cavity of the patient showing missing teeth is illustrated in Figure 2. The oral cavity of the patient showing peg-shaped teeth is shown in Figure 3. The fingernails of the patient are demonstrated in Figure 4.

## Discussion

Ectodermal dysplasia has two types, namely, hypohidrotic and hydrotic ectodermal dysplasia. Hypohidrotic ectodermal dysplasia (Christ-Siemens-Touraine syndrome) has a classical triad of hypodontia, hypotrichosis, and hypohidrosis [4]. However, hidrotic ectodermal dysplasia (Clouston syndrome) affects teeth, hair, and nails. In both, sweating is normal, i.e., sweat glands are not affected [4]. Oral cavity examination reveals missing permanent teeth, maxillary or mandibular hypodontia, and conical-shaped or peg-shaped incisors and canines may be observed [4,5]. Patients with hypohidrotic ectodermal dysplasia may face social anxiety or low self-esteem due to dental appearance.

Patients with hypohidrotic ectodermal dysplasia may suffer from pulmonary infection, failure to thrive, and hyperthermia [4,5]. Other skin manifestations such as mottled skin, café-au-lait spots, reticular hyperpigmentation, follicular hyperkeratosis, and vitiligo patches may be observed [5]. Oral cavity radiographs such as an orthopantomogram can be helpful [6]. Dental prostheses can improve dental cosmetic appearance [6]. The best management strategy can be designed based on an assessment of the socioeconomic condition of the patient as 150 variants of ectodermal dysplasia have been demonstrated. Pediatric dentists play a main role in the identification, management, and follow-up of these patients. Mutations in the *EDA*, *EDAR*, or *EDARADD *gene are responsible for preventing normal interaction between the ectoderm and the mesoderm [7]. Therefore, due to this mutation, the development of ectoderm-derived structures such as hair, sweat glands, and teeth is impaired leading to hypohidrotic ectodermal dysplasia [7]. A prenatal diagnosis is made on fetal skin biopsy. A sample of skin biopsy obtained by fetoscopy at 20 weeks gestation and histopathological findings correlate with a lack of pilosebaceous follicles [8]. A multidisciplinary team approach is an appropriate strategy to manage these patients with the clinical knowledge of growth development and behavior management according to the patient’s needs [8]. Psychological well-being can be ensured by consultation with a psychologist, and psychological counseling can be advised if needed. Phonetics or articulation problems are detected through consultation with a speech therapist and an otolaryngologist [8].

## Conclusions

We report this case because of a rare association between hypohidrotic ectodermal dysplasia and idiopathic finger clubbing with a normal chest X-ray. Management is multidisciplinary including pediatricians to manage and monitor acute complications such as hyperpyrexia. Dentists look after dentures and prosthetics. Nutritionists can access and correct nutritional deficiencies if needed. Psychologists ensure the psychosocial well-being of children. Dermatologists evaluate skin conditions such as dry skin and eczema, and physicians monitor the general health of patients with ectodermal dysplasia.
